# Blended learning for postgraduates; an interactive experience

**DOI:** 10.1186/s12909-019-1717-5

**Published:** 2019-07-30

**Authors:** Mirjam Westerlaken, Ingrid Christiaans-Dingelhoff, Renée M. Filius, Bas de Vries, Martine de Bruijne, Marjel van Dam

**Affiliations:** 1 Elevate Health, Utrecht, The Netherlands; 2 Amstel Academy, VU University Medical Center, Amsterdam, The Netherlands; 3 Head of Education Affairs, Utrecht University, Utrecht, The Netherlands; 4 Department Quality of Care and Patient Safety, University Medical Center Utrecht, Utrecht, The Netherlands; 5 Department of Public and Occupational Health, Amsterdam Public Health Research Institute, VU University Medical Center, Amsterdam, The Netherlands; 6 Intensive Care Center and Center for Research and Development of Education, University Medical Center Utrecht, Heidelberglaan 100, 3584 CX Utrecht, The Netherlands

**Keywords:** Social interactive learning, Postgraduate education, Health professionals, Blended learning, Deep learning

## Abstract

**Background:**

Blended learning has the potential to stimulate effective learning and to facilitate high quality education. For postgraduate health professionals, blended learning is relatively new. For this group we developed, implemented and evaluated two blended learning modules in a master program on quality and safety in patient care. Aiming for a better preparation compared to traditional textbook homework, the course provided not only web lectures and reading, but also interactive assignments and collaborative learning. Additional goal was saving time for the teachers resulting in a potential cost savings.

**Method:**

The experiences of 21 postgraduate health professionals were evaluated with two voluntary and anonymous questionnaires beginning of 2017 with a special focus on the added value of online interaction, underexposed in previous research.

**Results:**

This evaluation shows that online modules are regarded as being an effective preparation for face-to-face meetings for postgraduate health professionals. Added value of social interactive online preparation was perceived from collaborating and interacting with each other. Both the interaction between the students, and the e-moderator and teachers were well received.

**Conclusions:**

Based on the results of this study, we suggest that blended learning may indeed increase the level of education and stimulate effective learning for postgraduate health care professionals. The professionals experienced added value of social interactive online preparation from collaborating and interacting with each other. We consider better aligning of the online and face-to-face components as one of the highest priorities.

**Electronic supplementary material:**

The online version of this article (10.1186/s12909-019-1717-5) contains supplementary material, which is available to authorized users.

## Background

Blended learning is the integration of an online and face-to-face learning experience. In the last decades, blended learning has been getting more and more importance in academic education [1]. It not only shifts the education from teacher-centered to student-centered, but also has the potential to increase interaction between students and teachers, leading to improved learning [2]. Online learning as a preparation for face-to-face educational meetings has many advantages: nowadays it is easily accessible at any place and any time, students can learn at their own pace and start and follow the course as desired. In addition blended learning contributes to students’ motivation and satisfaction [3] and stimulates a feeling of autonomy and responsibility [4]. After the development and implementation it can be deployed frequently for multiple target groups, which is beneficial from a financial point of view as well.

Also for health professionals, blended learning has the potential to improve clinical competencies [5]. Liu et al. compared the effectiveness of blended learning to traditional face-to-face and fully online education for health professions. They cautiously concluded that blended learning contributes more effectively to knowledge aqcuisition than face-to-face and fully online education [6]. Main reasons seems to be that participants can review educational materials whenever and wherever they want [7] and that a feeling of loneliness or lost of interest in subject matter is being prevented by also meeting face-to-face [8]. One blended approach that is often used is the ‘Flipped classroom’ concept in which face-to-face teacher instructions are replaced with individual or group homework activities [9]. A recent meta-analysis on the ‘Flipped classroom’ concept in health professionals showed improved student learning compared with traditional teaching methods [10].

Blended learning for postgraduate health professionals is relatively new as well as the focus on social interactive component of online learning. The added value of the social interactive component in blended approaches is of great importance to facilitate effective and deep learning [8, 11]. Also, the social constructivist theory emphasizes the importance of social interaction between students to students and teachers in order to stimulate effective learning [12]. Participants should be given opportunity to construct and gain knowledge by using active learning methods and exchanging views. Social interaction and collaborative learning online stimulate the development of critical thinking skills, the co-creation of knowledge and meaning, reflection and transformative learning [13]. More knowledge about the benefits and challenges of social interaction online within a blended program, could optimize online interaction for postgraduate health professionals.

### Research question

This paper describes the introduction and evaluation of a blended learning approach for educating health professionals. The design and exploitation of our program is founded on the social constructivist theory, emphasizing the importance of social interaction in order to stimulate effective learning. Therefore, we will reflect on the question: *What is the added value of online social interaction in blended approaches for postgraduate health professionals?*

The blended approach invites students to interact and discuss the learning content not only 2 days a month during the face-to-face meetings but also during the modules online. As interaction is often considered as a prerequisite for deep learning [14] we expected the learning effect for students to increase. Because participants study parts of the learning content already online and through social interaction among peers with little support from teachers, it was expected that face-to-face teaching time for faculty would diminish.

## Method

This article describes the evaluation of the implementation of a newly developed blended learning concept for postgraduates. We describe the development, implementation and evaluation of blended learning for postgraduates and the added value of social interaction.

### Development

The Netherlands Federation of University Medical Centers or NFU, representing eight cooperating University Medical Centers in The Netherlands offers a master program for health care professionals “Quality and safety in patient care”. The multidisciplinary group of 21 postgraduates consisted of 7 men and 14 women, aged 28 to 58 years, with different backgrounds: ten nurses, eight medical doctors, one pharmacist, one paramedic and one policy officer. All health professionals are directly or closely involved in patient care and combine a fulltime job with this master program.

The NFU master program originally has the same classical structure as many other educational programs in higher education: individual passive homework activities, mainly reading articles, followed by face-to-face meetings with lectures and extensive interaction. The redesigned blended model with a focus on social interaction online intended to increase effective learning for post academic health professionals working in hospitals by replacing passive homework activities like reading articles with interactive online learning activities. An additional goal would be diminishing the teaching time for teachers by replacing some face-to-face elements through online learning activities with a lot of peer to peer interaction.

In the fall of 2016 two blended modules for postgraduate health professionals on patient safety in hospitals have been developed and implemented according to the social constructivist theory. In both modules the importance of a safe culture, risk awareness and behavior within health care were accentuated. The first module offered an introduction to crew resource management and the incident analysis in theory and practice. The second module offered an introduction in scientific research on patient safety and the legal consequences of a patient safety incident.

### Implementation

After following five traditional face-to-face modules of the masterprogram, two blended modules were introduced in January and February 2017 according to the ‘Flipped classroom’ concept. Both blended modules started with 2 weeks preparation online, containing individual learning activities without interaction (web lectures, video’s, readings), individual learning activities including interaction with the computer (polls, quizzes) and collaborative activities including interaction with peers and faculty (discussion forums, peer review assignments, wiki’s). Since this paper intends to evaluate the added value of social interaction, we focus on the third category of activities. The first module consisted of 3 web lectures, 8 video’s, 8 reading activities, 3 quizzes, 8 discussion forums, 1 peer review assignment, 2 wiki’s and 1 poll. The second module consisted of 3 web lectures, 6 video’s, 13 reading activities, 6 quizzes, 5 discussion forums, 3 peer review assignment, 4 wiki’s and 3 polls. More information about the structure of the online modules can be found in Additional file 1.

The online components were offered through the virtual learning environment, a Moodle-based platform, and contained asynchronous activities. The online activities opened to all participants at the same time and closed after 2 weeks the same way. During these weeks participants could complete the online activities in their own pace. Both weeks one topic was addressed, and at the end of the weeks students were asked to pose remaining questions online. The 2 weeks of online preparation were followed by 2 days of face-to-face education. During these days, the remaining questions posed online were discussed. In addition time was spent to practice what was learned online with real patients situations, for example disclosure procedures.

Since faculty plays an important role in facilitating and stimulating interaction between participants during online education [15], the online activities were taught by an e-moderator (with a MSc in Educational Sciences) and four content experts from the NFU (one teacher per week, two for each module). The e-moderator had initial contact with students, was online every day to check for questions, sent friendly reminders and weekly updates, resolved practical and technical issues and contacted teachers about content-related questions. The teachers provided feedback, answered content-related questions and provoked discussions by asking relevant questions. All for the purpose of effective and higher order learning [16].

### Evaluation

To explore students’ experiences, both blended modules were evaluated with an online questionnaire about the online learning activities and a questionnaire on the face-to-face component. Each participant received a questionnaire after completing a module.

In the questionnaire concerning the online components participants were asked to rate the learning materials, the supervision of faculty and the interaction with peers. The questionnaire contained five rating questions regarding the collaborative activities, five rating questions regarding the e-moderator and four rating questions regarding the teachers. The questions regarding the collaborative activities focused on the quality and frequency of the contributions, the availability of an expert, the encouragement to contribute actively and the use of a discussion forum as a tool for learning. The questions regarding the e-moderator focused on the quality of the messages and help, the encouragement, the speed of response and the need for an e-moderator. The questions regarding the teachers focused on the amount and quality of feedback, the content expertise and the speed of response. An example question for each topic is stated in Table 1.

**Table 1:** Example questions regarding the collaborative activities, e-moderator and teachers

The collaborative activities	How do you rate the quality of the contributions to the discussion forums?
The e-moderator	How do you rate the encouragement from the e-moderator?
The teachers	How do you rate the quality of the feedback by the teachers?

For all rating questions, a 5-point Likert scale, from 1 (lowest) to 5 (highest), was used. At the end of all three topics participants could give suggestions in open-ended questions. Additionally 4 open ended questions were asked concerning expectations, what participants specifically liked, technical problems encountered and suggestions for improvement.

The questionnaire used for evaluating the experiences has been developed previously and has not been published elsewhere. An English language version is available as Additional file 2. From previous data from 2016 a reliability analysis was performed in SPSS 23.0 for Mac OS. This analysis showed that the questionnaire had a high reliability: Cronbach’s alpha of the constructs ‘collaborative activities’, ‘e-moderator’ and ‘teacher’ were respectively .837 (*n* = 423, five items), .886 (*n* = 415, five items) and .871 (*n* = 414, five items). To extend the value of the evaluation results, from 2017 one item, ‘The speed of the response of the teacher’, has been added to the questionnaire. This has not negatively influenced the reliabilty of the construct ‘teacher’. Cronbach’s alpha of the constructs ‘collaborative activities’, ‘e-moderator’ and ‘teachers’ for this study were respectively .610 (*n* = 32, five items), .792 (*n* = 32, five items) and .869 (*n* = 32, four items).

The questionnaire concerning the face-to-face meeting invited the students to give comments or suggestions on the face-to-face components anonymously and voluntarily and the purpose of the questionnaires and how the results would be analysed was explained. The questionnaires concerning the online and offline components were completed by 16 and 15 students, respectively. The data from both questionnaires were analysed and reported in two evaluation reports. In The Netherlands evaluation questionnaires regarding newly introduced educational programs are not submitted to ethical approval by Institutional Review Boards. The purpose of the evaluation questionnaires was explained to the participants. Their participation was anonymous and voluntary. The obtained data were not traceable to participants.

## Results

Results of this study indicate that blended learning with interactive learning methods may increase the perceived learning results and overall satisfaction. The participants rated the online modules overall 7.6 median on a scale from 1 to 10, with range 7–9 for the first online module and range 6–9 for the second online module.

In the first online module, the study load was rated as ‘just enough’ by all responders (*n* = 14). In the second online module (responders *n* = 16) the study load was rated ‘too little’ by one responder, ‘just enough’ by 13 responders and ‘too heavy’ by two responders. The learning environment, the usability and speed, were marked 7.5 median on a scale from 1 to 10.

### The collaborative activities

Participants were moderately positive about the interaction with peers and faculty online. Table 2 shows the scores students assessed the discussion forum with. These scores are similar to other online courses offered by Elevate in 2017 [17].

**Table 2:** Students’ assessment of the discussion forums on a scale from 1, lowest, to 5, highest (mean score)

The quality of the contributions to the discussion forum	3.8
The frequency of contributions to the discussion forum	3.4
The availability of an expert that added comments to the discussion forum	3.7
The encouragement to actively contribute to the discussion forum (if needed)	3.5
The use of a discussion forum as a tool for learning	3.6

On the question if students have any suggestions regarding the discussion forum, ten students commented with a positive remark about the value of discussion forum, e.g.

“*I like this way of learning together.”*

*“Valuable to discuss with each other.”*

Also ten students commented with a negative comment about the value of the discussion forums. Three disadvantages mentioned are the amount of comments which was experienced as overwhelming by some participants, some technical limitations, and the obligatory character of the discussion forums.

*“I think the discussion forum was a nice feature, but might have been used too often. Especially when it required commenting on another participant's post, this could actually be a limitation in the ‘freedom of learning’.”*

*“The concept is super, but two big disadvantages: too many participants, overwhelming amount of messages. (…) And I missed a parent tree, (…), but did not find it easy to categorise discussion around assignment.”*

### The interaction with the e-moderator

Regarding supervision online, students’ assessment of the e-moderator is stated in Table 3.

**Table 3:** Students’ assessment of the e-moderator on a scale from 1, lowest, to 5, highest (mean score)

The quality of the messages from the e-moderator	3.7
The encouragement from the e-moderator	3.5
The quality of the help by the e-moderator	3.8
The speed of the response of the e-moderator	4.1
The need for an e-moderator	3.4

Students appreciated that support was available whenever they had questions or experienced problems. Also the weekly updates were appreciated. In total, ten positive remarks about the moderator were made.

*“I really liked the emails as a reminder*.”

*“It was nice that there is someone to answer possible questions or uncertainties, and that she is proactive.”*

Five students commented with a negative comment on the interaction with the e-moderator. Especially in the first module some participants thought that the messages of the moderator were a little strict.

*“I think that it’s good that you kept your finger on the pulse. I just felt like I was treated a little childish when I had to explain in the first week during my vacation why I had not logged in yet. We are all adults and it is our own responsibility.”*

### The interaction with the teachers

The quantitative results regarding the teachers are shown in Table 4.

**Table 4:** Students’ assessment of the teachers on a scale from 1, lowest, to 5, highest (mean score)

The amount of feedback by the teacher(s)	3.6
The quality of the feedback by the teacher(s)	3.8
The content expertise of the teacher(s)	3.9
The speed of the response of the teacher(s)	3.9

Participants liked that they had interaction with their teachers before the face-to-face meeting. Three students gave a positive comment regarding the teachers, for example:

“*I liked that they reacted on the topics. There is already contact before the face-to-face meeting, for me that really is of added value.”*

Two participants disliked that the teacher did not react on all comments, and that they found it sometimes hard to find teacher’s feedback.

*“I wonder why the teachers sometimes replied and sometimes not. For me it seemed that replies were posted randomly. I would choose one approach so that it’s clear for all students when suggestions from teachers can be expected.”*

### The value of online social interaction in general

All participants answered the questions “What did you specifically like about this online component?” Nine out of 16 participants mentioned that they mostly liked the interaction with peers and faculty. Participants commented that they liked to interact and preferred learning together above the individual passive readings which was their homework for the other modules. They mentioned that interacting with peers and teachers online was beneficial for their learning process.

*“More guidance and interaction than when you’re reading alone. It feels less like doing it ‘on your own’. And it’s possible to give and get feedback from peers immediately*.”

*“It’s great to see that peers add new stuff. Therefore, this had added value above preparation alone.”*

*“I liked seeing the progress of my peers. Normally you’re struggling as a student ‘on your own’ to finish your homework and now it felt like we were doing it as a group.”*

*“I learned a lot from the activities about my own research. Reading feedback from my peers was useful and gave me new ideas.”*

### The alignment between the online and offline component

According to participants the quality of alignment differed per module. When asked for comments or suggestions, three students commented that the alignment could have been better.

*“I learned a lot from reading literature and completing the online learning activities. Unfortunately, the face-to-face meetings didn’t align. This did not meet my expectations: at the end of the e-learning you could ask questions and mention subjects you’d like to learn more about or deepen, but this wasn’t referred to.”*

*“I think that the e-learning was a pleasant way to prepare for the face-to-face meetings. (...) Blended learning aims to deepen the content during the face-to-face meetings. I think this didn’t work out very well during the two modules.”*

*“The alignment between the online and face-to-face component can be more tight.”*

For one presentation of the face-to-face component, teachers activated participants’ prior knowledge by directly referring to the questions students asked at the end of the online preparation, which was much appreciated.

## Discussion

### The added value of social interaction in blended approaches

This paper intended to evaluate the added value of social interaction in blended approaches for postgraduate health professionals. Overall, the results of this study suggest that, according to students, blended learning consisting of online preparation and face-to-face meetings is an effective educational strategy for postgraduate students. This was a highly motivated group of postgraduate students, who take ownership of their learning while combining a fulltime job with this master program. That is one of the reasons a flexible preparation of the online courses is well-received.

According to the students the online learning activities are a better preparation for the face-to-face meetings compared to the traditional, more passive homework. Students valued the social interactions through collaborative preparation online, but also the practical application of the online learning activities. These findings confirm our expectations that online social interaction stimulates students’ motivation more than traditional homework. Participants preferred to have discussions in smaller groups with a less obligatory character. Brindley, Walti and Blaschke [18] showed that smaller groups, consisting of four to six students, contribute to the effectiveness of collaborative learning, while Uijl et al. [11] confirmed that in higher education, obligatory discussions do not contribute to more effective interaction than non-obligatory discussions. Our recommendation is to include small groups with non-obligatory discussions in the online learning activities.

Important limitations of this evaluation are the small sample size, the lack of a control group, only one e-moderator and a small number of teachers. Consequently, our experience is not immediately generalizable. Also the questionnaire is confined, but validated and all items are discussed in the Result section. Another limitation of this project is that the evaluated group of mixed health professionals has a high level of ambition, combining a fulltime job with this master program. However, we do consider the results generalizable to other postgraduate students who choose consciously for their professional development. The absence of structural evaluation of the experiences of the teachers is also a limitation. Informally, the new program was assessed with the teachers after the face-to-face meetings by two of the authors (MvD and MdB). All of them agreed that developing and implementing a new blended learning course is time-consuming. This has been shown by other authors as well, e.g. Alebaikan et al. [19], Filius et al. [20] and Kenney et al. [21]. It did not, against expectations, diminish the time needed for face-to-face meetings as Garrison and Vaughan [10] stated in their book. On the one hand, this had to do with the social interaction of the group: participants value their two-days of face-to-face education very highly. On the other hand, according to teachers, the newly, mostly online acquired knowledge of the participants, took the level of education to a higher standard. Participants gave more input which led to more meaningful interaction. The teachers could focus more on application of the acquired knowledge by the students rather than explaining it. In the presence of real patients, available during the face-to-face meetings, this may contribute even more to professional development of postgraduates [22]. Similar to Stockwell et al. [23] this indicates that blended learning could lead to deep learning skills as students are aiming for understanding as apposed to surface learning where they are aiming to memorize or reproduce material for a test [24, 25].

### Teaching experiences

Teaching a blended course differs from traditional face-to-face teaching in a way that it is challenging to move from the structured learning methods approach to a more open and deepening face-to-face discussion. That corresponds with the participants’ comments that the alignment between the online and face-to-face components could be improved. Teaching blended requires a different approach for teachers. Other studies have confirmed this result as well [19–21, 26, 27]. Teachers should get support and education on how to adapt their teaching style to a blended approach. Including educational experts in the development and implementation of a blended learning program can help to align better. One practical suggestion for teachers is to activate participants’ prior knowledge, which will contribute to effective learning [12, 22] and deep learning [25, 28]. When asking participants to pose remaining questions online and to share topics they would like to discuss further, it is crucial that these items are addressed during the face-to-face meeting in order to activate prior knowledge and keep students motivated.

## Conclusion

Based on the results of this study, we suggest that blended learning may indeed increase the level of education and stimulate effective learning for postgraduate health care professionals. The professionals experienced added value of social interactive online preparation from collaborating and interacting with each other. It is recommended to facilitate social interaction online with small discussion groups.

Both the interaction between the students and the e-moderator and teachers were well received. Better aligning the online and face-to-face components we consider as one of the highest priorities developing blended learning programs. The development and implementation of blended learning in this master program did not diminish the need for face-to-face meetings. For better aligning the online and face-to-face components, teachers should get support how to adapt their teaching style to a blended approach.

## Additional files

Additional file 1: **Table S1.** Design of the learning activities of the first online module. **Table S2.** Design of the learning activities of the second online module. (DOCX 17 kb)

Additional file 2: English version of the evaluation questionnaire. (DOCX 17 kb)

## Data Availability

The data that support the findings of this study are available per request from Elevate Health or from the corresponding authors.

## Abbreviations

BV: Bas de Vries
ICD: Ingrid Christiaans-Dingelhoff
MdB: Martine de Bruijne
MvD: Marjel van Dam
MW: Mirjam Westerlaken
NFU: Netherlands Federation of University Medical Centers
RF: Renée Filius
