# Supplementary material for: Blended learning for postgraduates; an interactive experience
Source: BMC Med Educ. 2019 Jul 30;19:289. doi: 10.1186/s12909-019-1717-5 (PMC6664728; doi:10.1186/s12909-019-1717-5)
Supplement: Supplementary file 1 — Table S1. Design of the learning activities of the first online module. Table S2. Design of the learning activities of the second online module. (DOCX 17 kb) [file 12909_2019_1717_MOESM1_ESM.docx]

Table S1. *Structure of learning activities first online module.*

| **Topic** | **Type of learning activity** | **Interaction** | **Study time in minutes** |
| --- | --- | --- | --- |
| Introduction | Video | None | 5 |
| Introduction | Reading | None | 5 |
| Introduction | Video | None | 5 |
| Introduction | Discussion forum | With peers and teachers | 20 |
| CRM in theory | Reading | None | 3 |
| CRM in theory | Web lecture | None | 5 |
| CRM in theory | Discussion forum | With peers and teachers | 10 |
| CRM in theory | Poll | With computer | 5 |
| CRM in theory | Discussion forum | With peers and teachers | 15 |
| CRM in theory | Web lecture | None | 2 |
| CRM in theory | Reading | None | 10 |
| CRM in theory | Quiz | With computer | 8 |
| CRM in theory | Web lecture | None | 15 |
| CRM in practice | Reading | None | 3 |
| CRM in practice | Discussion forum | With peers and teachers | 10 |
| CRM in theory | Video | None | 10 |
| CRM in practice | Quiz | With computer | 10 |
| CRM in theory | Video | None | 10 |
| CRM in theory | Wiki | With peers and teachers | 20 |
| CRM in practice | Video | None | 10 |
| CRM in practice | Reading | None | 10 |
| CRM in practice | Discussion forum | With peers and teachers | 10 |
| CRM in practice | Quiz | With computer | 5 |
| CRM in practice | Discussion forum | With peers and teachers | 5 |
| Incident Analysis | Reading | None | 3 |
| Incident Analysis | Video | None | 5 |
| Incident Analysis | Video | None | 22 |
| Incident Analysis | Video | None | 10 |
| Incident Analysis | Discussion forum | With peers and teachers | 10 |
| Incident Analysis | Reading | None | 25 |
| Incident Analysis | Wiki | With peers and teachers | 20 |
| Incident Analysis | Reading | None | 25 |
| Incident Analysis | Peer review | With peers and teachers | 20 |
| Incident Analysis | Discussion forum | With peers and teachers | 5 |

Table S2. *Structure of learning activities second online module.*

| **Topic** | **Type of learning activity** | **Kind of interaction** | **Study time in minutes** |
| --- | --- | --- | --- |
| Introduction | Video | None | 5 |
| Introduction | Reading | None | 5 |
| Introduction | Video | None | 5 |
| Introduction | Discussion forum | With peers and staff | 20 |
| Patient safety & study design | Reading | None | 3 |
| Patient safety & study design | Peer review | With peers and teachers | 30 |
| Patient safety & study design | Reading | None | 10 |
| Patient safety & study design | Poll | With computer | 3 |
| Patient safety & study design | Discussion forum | With peers and teachers | 10 |
| Patient safety & study design | Web lecture | None | 10 |
| Patient safety & study design | Quiz | With computer | 5 |
| Patient safety & study design | Web lecture | None | 10 |
| Patient safety & study design | Quiz | With computer | 5 |
| Patient safety & study design | Reading | None | 15 |
| Patient safety & study design | Quiz | With computer | 20 |
| Patient safety & study design | Peer review | With peers and teachers | 25 |
| Measurement & outcomes | Reading | None | 10 |
| Measurement & outcomes | Quiz | With computer | 25 |
| Measurement & outcomes | Discussion forum | With peers and teachers | 10 |
| Measurement & outcomes | Web lecture | None | 15 |
| Measurement & outcomes | Peer review | With peers and teachers | 30 |
| Measurement & outcomes | Discussion forum | With peers and teachers | 5 |
| Incidents | Reading | None | 3 |
| Incidents | Video | None | 3 |
| Incidents | Poll | With computer | 2 |
| Incidents | Quiz | With computer | 10 |
| Legal aspects | Wiki | With peers and teachers | 15 |
| Legal aspects | Reading | None | 2 |
| Legal aspects | Reading | None | 3 |
| Legal aspects | Video | None | 2 |
| Legal aspects | Wiki | With peers and teachers | 10 |
| Legal aspects | Reading | None | 2 |
| Legal aspects | Poll | With computer | 5 |
| Legal aspects | Quiz | With computer | 5 |
| Legal aspects | Reading | None | 5 |
| Dissatisfaction | Video | None | 3 |
| Dissatisfaction | Wiki | With peers and teachers | 17 |
| Dissatisfaction | Reading | None | 5 |
| Dissatisfaction | Reading | None | 12 |
| Dissatisfaction | Wiki | With peers and teachers | 20 |
| Dissatisfaction | Reading | None | 5 |
| Dissatisfaction | Video | None | 3 |
| Dissatisfaction | Discussion forum | With peers and teachers | 5 |
