# Supplementary material for: Blended learning for postgraduates; an interactive experience
Source: BMC Med Educ. 2019 Jul 30;19:289. doi: 10.1186/s12909-019-1717-5 (PMC6664728; doi:10.1186/s12909-019-1717-5)
Supplement: Supplementary file 2 — English version of the evaluation questionnaire. (DOCX 17 kb) [file 12909_2019_1717_MOESM2_ESM.docx]

**Evaluation Question Template**

Specific questions on the course:

1. Give an overall mark for the course, on a scale from 1 to 10.
2. How do you rate the content of the web lectures? (--, -, +-, +, ++)
3. How do you rate the technical quality of the web lectures? (--, -, +-, +, ++)
4. How do you rate the quality of the assignments? (--, -, +-, +, ++)
5. How do you rate the additional resources? (--, -, +-, +, ++)
6. How do you rate the final assignment/exam as a good reflection of the course contents. (--, -, +-, +, ++)
7. Do you have any suggestions regarding the learning materials? (open)
8. How do you rate the quality of the contributions to the discussion forum? (--, -, +-, +, ++)
9. How do you rate the frequency of contributions to the discussion forum? (--, -, +-, +, ++)
10. How do you rate the availability of an expert that added comments to the discussion forum? (--, -, +-, +, ++)
11. How do you rate the encouragement to actively contribute to the discussion forum (if needed)? (--, -, +-, +, ++)
12. How do you rate the use of a discussion forum as a tool for learning? (--, -, +-, +, ++)
13. Do you have any suggestions regarding the discussion forum? (open)
14. How do you rate the quality of the messages of the e-moderator? (--, -, +-, +, ++)
15. How do you rate the encouragement of the e-moderator? (--, -, +-, +, ++)
16. How do you rate the quality of the help of the e-moderator? (--, -, +-, +, ++)
17. How do you rate the speed of the response of the e-moderator? (--, -, +-, +, ++)
18. How do you rate the need for an e-moderator? (--, -, +-, +, ++)
19. Do you have any suggestions regarding the e-moderator? (open)
20. How do you rate the amount of feedback of the instructor(s)? (--, -, +-, +, ++)
21. How do you rate the quality of the feedback of the instructor(s)? (--, -, +-, +, ++)
22. How do you rate the content expertise of the instructor(s)? (--, -, +-, +, ++)
23. How do you rate the speed of the response of the instructor(s)? (--, -, +-, +, ++)
24. Do you have any suggestions regarding the instructor(s)? (open)
25. How do you rate the usability of the learning environment? (--, -, +-, +, ++)
26. How do you rate the speed of the learning environment? (--, -, +-, +, ++)
27. Give an overall mark for the learning environment, on a scale from 1 to 10.
28. Do you have any suggestions regarding the e-learning environment? (open)
29. What do you think of the study load? (too little, just enough, too heavy)
30. Please indicate how much hours you spent on Learning Unit 1 (drop down)
31. Please indicate how much hours you spent on Learning Unit N (drop down)

General questions on the course:

1. What were your expectations of the course before it started? (open)
2. Did the course meet your expectations? Please explain how it did or did not. (open)
3. What did you specifically like about this Elevate course? (open)
4. How likely is it that you would recommend Elevate to a friend or colleague, on a scale of 1 to 10?
5. How many times have you taken a distance learning course? (1^st^ time, 2-3 times, 4-5 times, more than 5 times)
6. Would you take a distance learning course again? (yes, no)
7. If not, please explain your answer. (open)
8. Did you experience any technical problems and if so, which one(s)? (open)
9. Do you have any suggestions for improvement? (open)
10. Your feedback is very valuable to us. We would like to contact some participants for more information about their experiences with the course. If you agree to participate in this, please enter your e-mail address.
